# Association between working in awkward postures, in particular overhead work, and pain in the shoulder region in the context of the *2018 BIBB/BAuA Employment Survey*

**DOI:** 10.1186/s12891-021-04482-4

**Published:** 2021-07-15

**Authors:** Julia Barthelme, Martha Sauter, Charlotte Mueller, Falk Liebers

**Affiliations:** 1 Charité-Universitaetsmedizin Berlin, Berlin, Germany; 2 Federal Institute for Occupational Safety and Health (BAuA), Noeldnerstr 40/42, 10317 Berlin, Germany

**Keywords:** Cross-sectional study, BLOSSFELD’s job classification, Occupational groups, Overhead work, Musculoskeletal disorders

## Abstract

**Background:**

Musculoskeletal disorders are the leading cause of work-related sick leave and incur substantial socioeconomic costs. With the aging of our society and employees, the problem is exacerbating, and prevention is becoming increasingly important. According to previous studies, exposure to awkward postures, such as overhead work, is associated with musculoskeletal problems.

**Objective:**

This study aimed to determine the current prevalence of employees who work in awkward postures, specifically overhead, stratified by age, gender and occupation in the context of the *2018 BIBB/BAuA Employment Survey* and to analyze associations between awkward working postures, in particular overhead work, and pain in the shoulder region.

**Method:**

The study is based on secondary data from the German *2018 BIBB/BAuA Employment Survey*. We have included 14,327 of the 20,012 employees aged < 67 years who work at least 35 h per week who took part in the survey. The classification of participants in occupational groups is based on the Blossfeld classification. The multivariate analysis was conducted by applying robust Poisson regression models adjusted block by block to obtain the relation between the self-reported frequency of working in awkward postures, in particular overhead work, and the occurrence of arm pain and neck and shoulder pain. Prevalence ratios (PR) are reported as effect estimates.

**Results:**

12.7% of participants indicated that they are often exposed to awkward postures at work; 5.0% stated they often performed overhead work. The majority of these employees worked in agricultural, unskilled and skilled manual occupations. The crude prevalence is 17.4% for arm pain and 48.4% for neck and shoulder pain. If subjects reported that they often performed overhead work, the risk of arm pain increased by 18% (PR 1.18, CI 1.04–1.34, final model).

**Conclusion:**

Working in awkward postures, especially overhead work, is a risk factor for upper extremity musculoskeletal disorders. The development of prevention strategies should focus on the workforce in agricultural, unskilled and skilled manual occupations.

**Supplementary Information:**

The online version contains supplementary material available at 10.1186/s12891-021-04482-4.

## Background

Musculoskeletal disorders (MSD) are a relevant topic in workplaces. They are the most common reason for sick leave in the workforce (Germany: 21.9% in 2018) and lead to tremendous costs (loss of production: €18.5 billion, loss of gross value added: €31.7 billion/2018 in Germany) [1]. Due to later retirement ages and demographic change, it is assumed that musculoskeletal disorders in workplaces will become an even more common problem in future [2]. Furthermore, many developed countries with high-income economies face an acute shortage of skilled employees in many industrial sectors. As a result, there is an interest in current data on the prevalence and risk factors of MSD in different settings and countries to guide preventive measures at work. There is a significant relation between disorders of the lower back and shoulders and high physical workloads. Information provided by the Federal Institute for Occupational Safety and Health (BAuA) shows that MSD affected a large percentage of employees at work in the last 12 months. Around 46% of employees reported lower back complaints; 48.5% of employees also reported neck and shoulder complaints [3].

Complaints of arm and shoulder and shoulder disorders are discussed as health effects of working in awkward postures, in particular of overhead work [4–13]. While some analyses focus on verifiable musculoskeletal injuries [13, 14], others investigate self-reported outcomes, such as pain and other symptoms [5]. There is evidence that MSD, especially in the shoulder region, are associated with working in awkward postures [4–8, 13–15]. Working in awkward postures means working in positions that deviate from neutral upright and comfortable standing or sitting positions. These working positions lead to changes in shoulder girdle and glenohumeral biomechanics like a decreasing subacromial space and increasing mechanical pressure on the supraspinatus tendon. Effects might be shoulder pain and less efficiently operating muscles [16, 17]. Thus, completing such tasks requires increased and long-lasting static muscle force. Working overhead, respectively working above shoulder level, is one awkward posture that is commonly seen in workplaces [18]. Regarding work performed in tiring or painful body postures, the European Agency for Safety and Health at Work (EU-OSHA) reports a range of between 43 and 46% of employees who are exposed to such conditions for at least a quarter of their working time across the EU-28 states between 2005 and 2014 [19]. Wærsted et al. 2020 [20] investigated in a systematic review the association and the exposure-response relationship between work above shoulder level and shoulder pain and disorders. Thirty-four publications with sufficiently rated quality, and prospective, cross-sectional, and case-control study designs have been included. In the studies, exposure and outcome were assessed with a wide range of methods, using both self-reported and objective measurements. In summary, the authors have reported limited evidence of an association between arm elevation at work and shoulder disorders. The evidence is moderate for severe arm elevation, in which the elbow is above the shoulder, and shoulder disorders.

Why is it worth adding evidence to this topic? One aspect is that Wærsted et al. 2020 [20] were only able to consider one case-control study from Germany [13].

Further information about the health situation of the German labor force is, however, available. The Federal Institute for Vocational Education and Training (BIBB) and the Federal Institute for Occupational Safety and Health (BAuA) perform regular employment surveys every 6 years. The *BIBB/BAuA Employment Surveys* are large-scale, representative surveys of the German workforce. Each of these surveys includes close to 20,000 subjects of working age, and their results are used to provide scientific policy advice. The self-reported frequency of working in awkward postures is just one item on a long list of physical working conditions [21–24] covered in the survey. During the *2006 BIBB/BAuA Employment Survey*, 14.3% of subjects reported that they often work in awkward postures; this number increased to 16.6% in 2012 [25]. Unfortunately, the wording of the question regarding work in awkward postures was not differentiated enough to be comparable to the earlier survey at an international level [26]. Therefore, the item was changed and extended in the most recent survey in 2018. Subjects who reported that they often worked in awkward postures were at least asked to define the type of posture in detail (kneeling, overhead work, and bending). In their review, Wærsted et al. 2020 [20] stated that work performed in an overhead posture is common in a wide range of professions; however, descriptions of the prevalence of job-specific exposure to overhead work is lacking.

This study therefore aims to provide data on the prevalence of work in awkward postures, in particular work in overhead postures, within occupational groups of the German population, using the *2018 BIBB/BAuA Employment Survey*. The second objective is to analyze the association between working in awkward postures, in particular working in overhead postures, and shoulder and arm pain.

## Methods

### Study design

The study is a secondary data analysis of the *2018 BIBB/BAuA Employment Survey*. The *BIBB/BAuA Employment Surveys* are regularly conducted, cross-sectional surveys initiated to provide information about the health status and working conditions of the German labor force. The first survey took place in 1979; since then, as many as seven surveys have been conducted. The obtained data is based on self-reported, subjective information [22]. Participants of the *2018 BIBB/BAuA Employment Survey* were recruited and interviewed by phone in the time between October 2017 and March 2018. The sample included German-speaking participants who were at least 15 years old, worked 10 h or more per week and were paid for this work. Trainees and persons undergoing vocational training were not included. A sample of 20,012 employed persons participated in the *2018 BIBB/BAuA Employment Survey* [22].

### Exposure and outcome

**Fig. 1:**
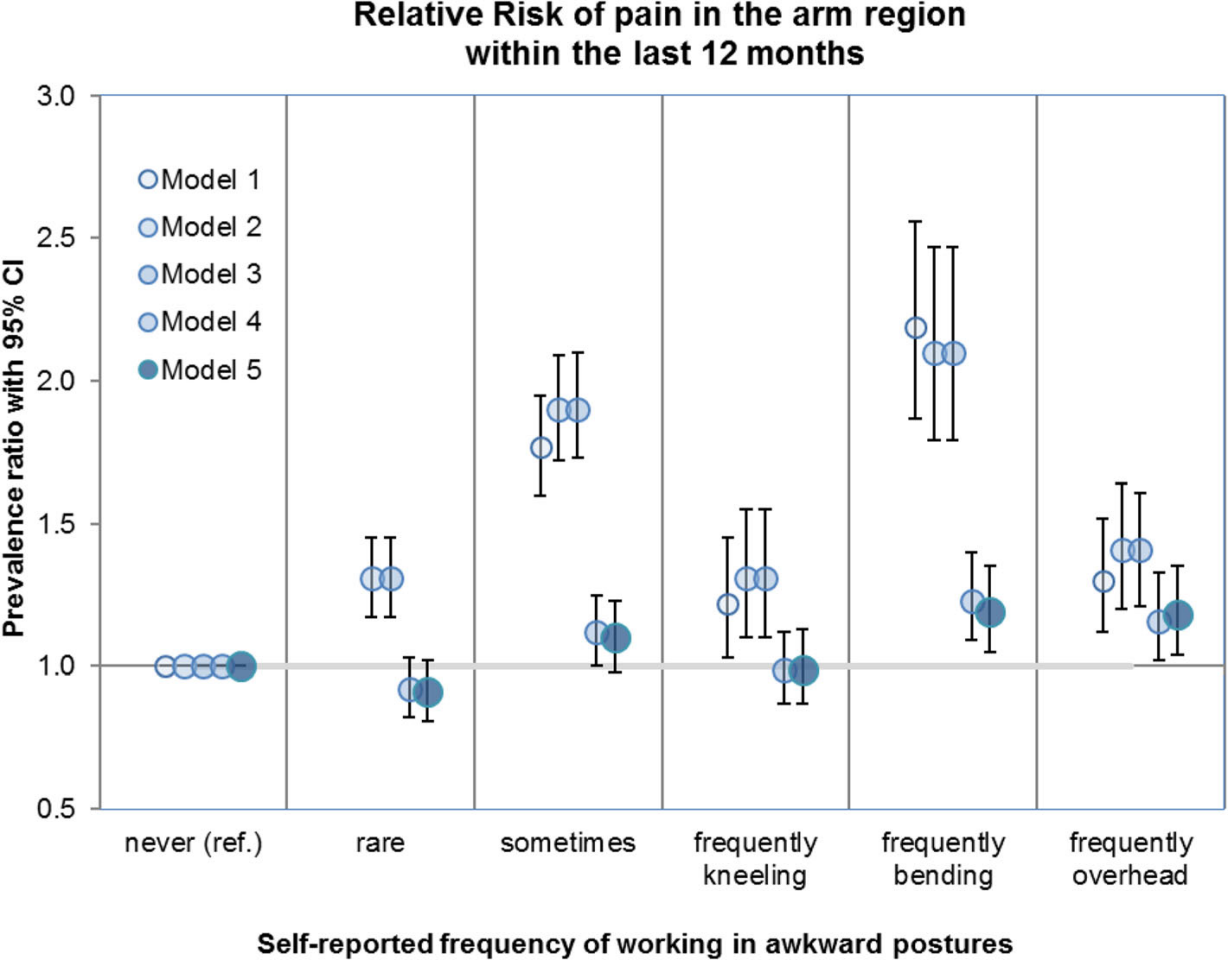
Relative risk of arm pain stratified by the frequency of working in awkward body postures

During the interview, participants were asked about the frequency of work performed in awkward postures. They could choose one of the following answer categories as a response: “never”, “rarely”, “sometimes”, and “often”. These four response categories referred to the job generally, but not to a specific frequency or duration per shift or per hour. If participants responded “often”, they were also asked if they often worked in an overhead posture, on their knees, or in a bended position. Possible answers were “yes” or "no”. Multiple responses that included all three postures were allowed. To include information about overhead work in the regression models, dummy variables were created. Further information about the construction of the dummy variables is provided in Figure 1 in the additional material.

Additionally, participants were asked about the occurrence of musculoskeletal pain or complaints in different body parts that occurred at work or after work in the last 12 months. No question explicitly focused on shoulder pain or disorders; we have therefore selected responses related to “pain/complaints in the arms” and “pain/complaints in the neck or shoulders” as outcomes. The options for answering these questions were “yes” or “no”.

### Covariables

In accordance with other studies [4, 5, 8, 11–13, 15, 18, 27–35], covariables were selected to reduce potential confounder bias in the analysis of the association between exposure and outcome. These covariates are gender (men, women), age (in years), other physical and environmental working conditions, weekly working time (hours) and the psychosocial workload. The occurrence of manual handling of heavy loads (“lifting or carrying of load >20 kg for men or >10 kg for women”), manual handling operations (“requiring high manual occupational skills, rapid manual movements and/or high manual forces”) and climatic workloads (“work in cold, heat, wet, damp or draft conditions”) were similarly gathered using the answer options “never”, “rarely”, “sometimes”, and “often”. To measure the psychosocial workload, we used an index based on a validated index introduced by Kroll in 2011 [36]. We have used three subcategories of the index (social burden, psychological, and temporal workload) to create a score (ranging from 0 to 100 index points) by adding the point values of the answered items and dividing them by the sum of the maximum values of answered questions. The higher the score, the greater the psychosocial workload [36].

### Preparation of data

Further exclusion criteria were applied to the study sample. We excluded people who worked on average (including overtime) less than 35 h per week in their main job as well as persons aged 67 or over.

Data were tabulated, inspected, examined for plausibility and checked for missing data. Secondary variables were built and, if necessary, the code of response categories was converted. All subjects (*n* = 14,327) who were included had no missing data in the outcome parameters and the selected covariates used in the regression analysis (complete-case-analysis). The resulting sample did not include minors under the age of 16 years.

### Data analysis

The number of participants, their age, weekly working hours, psychosocial workload, gender, arm pain, neck and shoulder pain, physical and climatic workload as well as the classification of the occupation were stratified by the frequency of work performed in awkward postures and overhead work. We have used absolute numbers (n) and prevalence (%) to describe the distribution of categorical variables. Arithmetic means and standard deviations (SD) were used to characterize numeric variables. Associations between exposure and binary outcome were estimated using Poisson regression models with robust variance estimates. Poisson regression was used to derive prevalence ratios (PR) directly from effect estimates. The robust variance estimates were exploited to achieve correct 95% confidence intervals (CI) and to control for over- and underdispersion [37]. Statistical analyses were performed using SPSS statistics 25 and are fully syntax based.

The associations between exposure and the two outcomes (arm pain and neck and shoulder pain) were examined by applying five models that were adjusted block by block for each outcome. Based on a literature search a list of relevant covariates was derived from reported associations between working in awkward postures, including overhead work, and shoulder pain [4, 5, 8, 11–13, 15, 18, 20, 22, 27–35]. This a-priori defined list of covariates was included in an underlying causal diagram and considered in the regression models [38]. The selected confounders were gradually added to the regression models block by block (Model #1: exposure only; Model #2: #1 with age and gender; Model #3: #2 with actual weekly working hours, Model #4: #3 with other physical workloads). Finally, fully adjusted Model #5 includes the following covariates: age, gender, actual weekly working hours, and manual handling of heavy loads, manual (repetitive) handling operations, climatic and psychosocial workload. This fully adjusted model (Model #5) has been defined as the model used for reporting and interpretation.

The adjusted prevalences of arm pain and neck and shoulder pain, stratified by the self-reported frequency of awkward postures, were derived by applying post-estimations based on the results of the final model. This was only applicable to the basic question regarding the frequency of work performed in awkward postures without this being split into the specific types of postures.

The occupations of participants in the *2018 BIBB/BAuA Employment Survey* were coded in accordance with German occupation classifications in the dataset. These coded job titles have been summarized to occupational groups, using the classification of job titles published by Blossfeld 1985 [39]. The Blossfeld classification system covers twelve groups: agricultural occupations, unskilled manual occupations, skilled manual occupations, technicians, engineers, unskilled services, skilled services, semiprofessions, professions, unskilled commercial and administrational occupations, skilled commercial and administrational occupations, and managers.

## Results

### Descriptive analysis

In total, 14,327 participants matched the inclusion and exclusion criteria and provided complete datasets regarding the variables used in the regression models.

The basic characteristics of the population used in the analysis are shown in Table 1. Due to missing data on job titles, the stratification by BLOSSFELD occupational groups includes only 14,265 subjects (Table 2). This dataset did not differ from the whole dataset of *n* = 14,327 participants regarding mean age, gender distribution, prevalence of shoulder and neck pain or arm pain, prevalence of exposure to awkward postures, and mean working time.

**Table 1:** Characteristics of the study population stratified by the frequency of working in awkward body postures (*n* = 14,327)

		Self-reported frequency of work performed in awkward postures					Work performed in specific awkward postures (percentages related to all participants)	
		Never	Rarely	Sometimes	Often	Total	Often performs overhead work	Often works in other awkward posture
Participants (n (row %)		8407 (58.7%)	2221 (15.5%)	1883 (13.1%)	1816 (12.7%)	14,327 (100%)	717 (5.0%)	1099 (7.7%)
Men (n (row %))		4808 (54.5%)	1567 (17.7%)	1293 (14.6%)	1162 (13.2%)	8830 (100%)	554 (6.3%)	608 (6.9%)
Women (n (row %))		3599 (65.5%)	654 (11.9%)	590 (10.7%)	654 (11.9%)	5497 (100%)	163 (3.0%)	491 (8.9%)
Women to men ratio (%)		42.8%	29.4%	31.3%	36.0%	38.4%	22.7%	44.7%
Age (mean (SD))		47.3 (10.9)	46.7 (11.0)	45.4 (11.5)	45.0 (11.7)	46.7 (11.1)	45.6 (11.6)	46.7 (11.2)
Weekly working hours (Mean (SD))		43.6 (7.0)	43.8 (7.5)	44.6 (9.2)	43.8 (8.2)	43.8 (7.6)	44.1 (8.3)	43.6 (8.3)
Psychosocial workload index by Kroll (mean (SD))		37.1 (10.9)	40.3 (12.0)	42.1 (12.4)	42.2 (13.4)	38.9 (11.8)	41.6 (13.1)	42.6 (13.5)
Arm pain (col %)	Yes	12.3%	16.4%	23.5%	36.0%	17.4%	39.6%	33.7%
Neck and shoulder pain (col %)	Yes	45.9%	45.9%	50.3%	60.6%	48.4%	58.7%	61.8%
Gender (col %)	Women	42.8%	29.4%	31.3%	36.0%	38.4%	22.7%	44.7%
	Men	57.2%	70.6%	68.7%	64.0%	61.6%	77.3%	55.3%
Manual handling operations (col %)	Never	66.5%	12.1%	9.0%	5.8%	42.8%	2.1%	8.2%
	Rarely	9.7%	28.9%	7.0%	5.1%	11.7%	3.8%	6.0%
	Sometimes	7.1%	20.6%	34.7%	11.6%	13.4%	11.0%	12.0%
	Often	16.7%	38.5%	49.3%	77.5%	32.1%	83.1%	73.8%
Manual handling of heavy loads (col %)	Never	70.2%	19.2%	14.9%	8.2%	47.2%	5.0%	10.3%
	Rarely	19.4%	44.7%	20.7%	13.8%	22.7%	13.1%	14.3%
	Sometimes	6.1%	17.0%	32.7%	16.5%	12.6%	16.3%	16.6%
	Often	4.3%	19.1%	31.7%	61.5%	17.5%	65.6%	58.9%
Climatic workload (col %)	Never	75.4%	31.1%	25.6%	21.3%	55.1%	11.9%	27.4%
	Rarely	9.9%	27.9%	12.3%	11.7%	13.2%	12.3%	11.4%
	Sometimes	8.7%	19.6%	34.5%	20.3%	15.3%	22.0%	19.2%
	Often	6.0%	21.4%	27.6%	46.7%	16.4%	53.8%	42.0%

Legend: row %: row percentage, n: absolute number, col. %: column percentage, SD: standard deviation

**Table 2:** Work performed in awkward postures stratified by gender in different occupational groups (*n* = 14,265 due to missing data in 62 cases)

BLOSSFELD occupational group	Never in awkward postures (row %)	Rarely in awkward postures (row %)	Sometimes in awkward postures (row %)	Often in awkward postures (row %)		Total (row %)	No. of subjects
				Often performs overhead work	Often works in other awkward postures		
**Men (*n*** **= 8786)**							
Agricultural occupations	9.1%	20.3%	35.3%	13.4%	21.9%	100%	187
Unskilled manual occupations	28.1%	23.9%	21.2%	11.7%	15.1%	100%	675
Skilled manual occupations	15.3%	18.8%	26.9%	23.3%	15.8%	100%	1210
Technicians	45.1%	24.5%	18.9%	6.2%	5.3%	100%	628
Engineers	71.5%	18.5%	7.8%	1.3%	0.9%	100%	769
Unskilled services	36.7%	26.9%	21.2%	8.2%	7.0%	100%	867
Skilled services	43.6%	22.4%	19.5%	4.4%	10.0%	100%	548
Semiprofessions	60.2%	16.9%	13.0%	0.5%	9.4%	100%	575
Professions	75.0%	14.4%	8.6%	0.4%	1.5%	100%	533
Unskilled commercial and administrational occupations	61.3%	14.4%	13.5%	2.3%	8.6%	100%	222
Skilled commercial and administrational occupations	80.7%	11.7%	5.9%	0.3%	1.4%	100%	1573
Managers	85.0%	9.2%	3.7%	0.7%	1.4%	100%	999
**All men**	**54.4**%	**17.8**%	**14.6**%	**6.3**%	**6.9**%	**100**%	8786
**Women (*n*** **= 5479)**							
Agricultural occupations	16.1%	16.1%	29.0%	11.3%	27.4%	100%	62
Unskilled manual occupations	50.0%	16.2%	19.5%	7.1%	7.1%	100%	154
Skilled manual occupations	44.2%	23.3%	15.3%	9.8%	7.4%	100%	163
Technicians	71.4%	15.1%	9.4%	2.1%	2.1%	100%	192
Engineers	79.6%	10.9%	6.6%	0.7%	2.2%	100%	137
Unskilled services	46.9%	21.4%	14.7%	6.3%	10.7%	100%	224
Skilled services	48.9%	19.8%	17.7%	3.7%	9.9%	100%	464
Semiprofessions	43.1%	13.1%	17.1%	4.7%	22.1%	100%	1419
Professions	77.7%	11.9%	6.3%	0.8%	3.4%	100%	528
Unskilled commercial and administrational occupations	60.7%	15.4%	13.2%	4.8%	5.9%	100%	272
Skilled commercial and administrational occupations	87.7%	6.3%	3.8%	0.6%	1.7%	100%	1333
Managers	92.5%	3.8%	2.4%	0.4%	0.9%	100%	531
**All women**	**65.4**%	**11.9**%	**10.8**%	**3.0**%	**9.0**%	**100**%	5479
**All men and women**	58.6%	15.5%	13.1%	5.0%	7.7%	100%	**14,265**

Legend: row %: row percentage). This dataset did not differ from the whole dataset of *n* = 14,327 participants regarding mean age, gender distribution, prevalence of shoulder/neck pain or arm pain, prevalence of exposure to awkward postures, and mean working time

12.7% of participants indicated that they “often” work in awkward postures (5.0% overhead and 7.7% in other awkward postures). 13.2% of men reported that they often work in awkward postures, compared to 11.9% of women. The mean age of participants who reported that they often perform overhead work is 45.66 years of age (SD 11.15). People who indicated that they often perform overhead work were also often exposed to manual handling operations or repetitive work (77.5%), manual handling of heavy loads (61.5%) and climatic workloads (46.7%) (Table 1). The mean psychosocial workload of subjects who often performed overhead work is higher than that of subjects who never worked in awkward postures (42.23 index points vs. 37.07 index points). (Table 1)

### Overhead work in various occupations

The majority of participants who reported that they often perform overhead work are employed in skilled manual occupations (men: 23.3%, women: 9.8%), agricultural occupations (men: 13.4%, women: 11.3%) and unskilled manual occupations (men: 11.7%, women: 7.1%). Table 2 shows the detailed distribution of self-reported exposure to awkward postures stratified by BLOSSFELD occupational groups and gender.

### Associations between overhead work and pain in the arm region

The unadjusted model (Model #1) and the adjusted models each found an association between persons who often perform overhead work and arm pain at work in the last 12 months. Effect estimates of Models #1 to #5 are presented in Fig. 1. The unadjusted model shows a higher prevalence ratio (PR 1.30, CI 1.12–1.52) than the fully adjusted model (PR 1.18, CI 1.04–1.34). The additional adjustment for other physical workloads in Models #3 and #4 leads to a considerable decrease in the prevalence ratio. However, the effect of overhead work in Model #5 remains significant.

After adjusting all considered covariables (Model #5, Table 3), the regression model shows an increase in the risk of arm pain from persons who never work in awkward postures (reference group) to those who responded “often perform overhead work” (PR 1.18, CI 1.04–1.34). For persons who responded “sometimes” the increase in the prevalence was only small and non-significant (PR 1.10, CI 0.98–1.23). The prevalence of arm pain does not increase in the category of those who “rarely” work in awkward postures. In comparison, among persons who reported that they never, rarely or sometimes worked in awkward postures, frequent overhead work is associated with an 18% higher prevalence of reporting of arm pain (Table 3). Besides overhead work, often performing work in bending postures was a risk factor for the occurrence of arm pain at work in the last 12 months (PR 1.19, CI 1.05–1.35). This effect is comparable to the effect of often performing overhead work. Frequent kneeling was not associated with arm pain.

**Table 3:** Prevalence Ratios with 95% confidence interval considering arm pain (*n* = 14,327)

	Prevalence ratios for arm pain (outcome) after adjusting for gender, age, weekly working hours and working conditions (Model #5)			
Age	Per year	1.020 (1.016–1.023)		
Gender	Women	1.454 (1.353–1.562)		
Weekly working hours	Per h	0.987 (0.982–0.992)		
Psychosocial workload (score)	Per unit	1.011 (1.008–1.014)		
	Never	Rarely	Sometimes	Often
Works in awkward postures	1 (ref.)	0.907 (0.809–1.016)	1.100 (0.984–1.230)	(answer split into 3 dummy variables)
1) Often performs overhead work	1 (ref.)	–	–	1.181 (1.037–1.345)
2) Often when bending	1 (ref.)	–	–	1.191 (1.050–1.351)
3) Often when kneeling	1 (ref.)	–	–	0.995 (0.874–1.132)
Manual handling of heavy loads	1 (ref.)	1.072 (0.957–1.200)	1.176 (1.033–1.339)	1.531 (1.359–1.724)
Manual handling operations	1 (ref.)	1.025 (0.879–1.195)	1.150 (1.001–1.320)	1.712 (1.534–1.911)
Climatic workload	1 (ref.)	1.063 (0.938–1.206)	1.296 (1.165–1.442)	1.646 (1.490–1.818)

Legend: ref. = reference group

The fully adjusted Model #5 provides supplementary information about the association of arm pain and the considered covariates: Other physical work factors such as the manual handling of heavy loads, manual handling operations and climatic workload are more strongly associated with arm pain than often performing overhead work (Table 3). When it comes to the psychosocial workload, a positive association with arm pain in the last 12 months was detected. Weekly working hours are negatively associated with self-reported arm pain. (Table 3). Women are more likely to report arm pain than men (PR 1.45, CI 1.35–1.56). Age is associated with an increased prevalence of reported arm pain (PR 1.22, CI 1.17–1.26, per 10 years).

Even without being split into specific work positions, working in awkward body postures is strongly associated with an increase in the prevalence of arm pain in the categories “sometimes works in awkward postures” (PR 1.17, CI 1.04–1.32) and “often works in awkward postures” (PR 1.36, CI 1.21–1.52) (Additional Table 1).

Based on the fully adjusted Model #5, and without splitting the category “often works in awkward postures” into specific work positions, the estimated prevalence of arm pain within the last 12 months is 21.6% (CI 19.7% - 23.6%) of arm pain in participants who often worked in awkward postures. The prevalence of arm pain for subjects who responded that they “sometimes”, “rarely” or “never” worked in awkward postures has been estimated at 18.6% (CI 16.9% - 20.4%), 15.2% (CI 13.8% - 16.7%) and 15.9% (CI 14.7% - 17.2%), respectively. The estimated prevalence was derived from the regression model presented in the additional material (Additional Table 1).

### Overhead work and pain in the neck and shoulder region

**Fig. 2:**
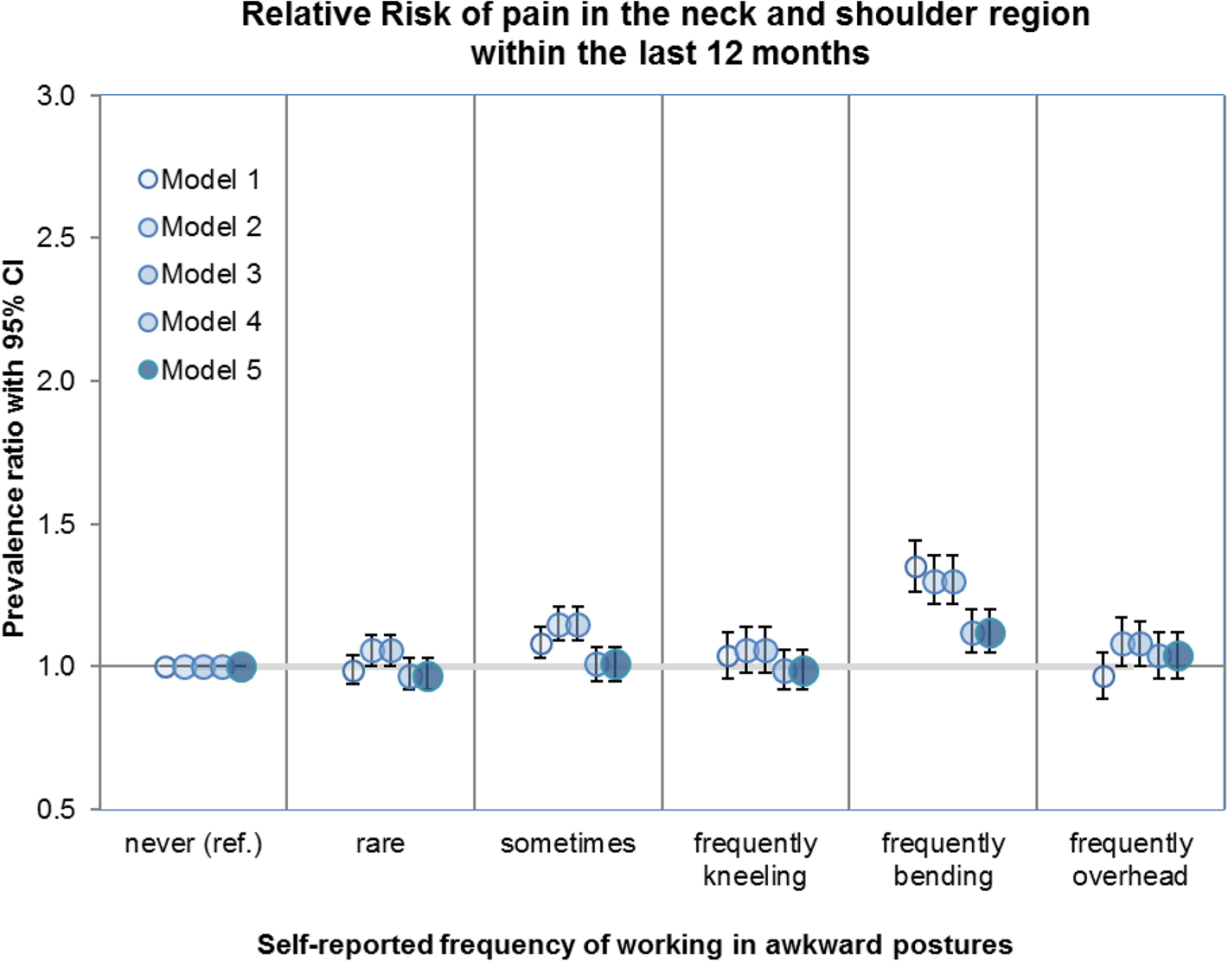
Relative risk of pain in the neck and shoulder region stratified by the frequency of working in awkward body postures

We have not detected any meaningful association between frequently performing overhead work and the other outcome of “neck and shoulder pain” in either the unadjusted model (Model #1: PR 0.97, CI 0.89–1.05) or the fully adjusted model (Model #5: PR 1.04, CI 0.96–1.12) (Table 4). The effect estimates for neck and shoulder pain based on Models #1 to #5 are presented as prevalence ratios in Fig. 2. The adjustment for other physical workloads in Model #3 leads to a considerable reduction in the prevalence ratio for neck and shoulder pain.

**Table 4:** Prevalence Ratios with 95% confidence interval considering pain in the neck and shoulder region (*n* = 14,327)

	Prevalence ratios for neck and shoulder pain (outcome) after adjusting for gender, age, weekly working hours and working conditions (Model #5)			
Age	Per year	1.003 (1.002–1.005)		
Gender	Women	1.571 (1.518–1.625)		
Weekly working hours	Per h	0.993 (0.990–0.995)		
Psychosocial workload (score)	Per unit	1.010 (1.009–1.012)		
	Never	Rarely	Sometimes	Often
Works in awkward postures	1 (ref.)	0.974 (0.922–1.029)	1.009 (0.953–1.068)	(answer split into 3 dummy variables)
1) Often performs overhead work	1 (ref.)	–	–	1.038 (0.960–1.122)
2) Often when bending	1 (ref.)	–	–	1.120 (1.047–1.199)
3) Often when kneeling	1 (ref.)	–	–	0.989 (0.922–1.060)
Manual handling of heavy loads	1 (ref.)	1.009 (0.961–1.060)	1.010 (0.951–1.072)	1.119 (1.058–1.184)
Manual handling operations	1 (ref.)	0.966 (0.906–1.029)	0.951 (0.895–1.011)	1.021 (0.973–1.071)
Climatic workload	1 (ref.)	1.006 (0.951–1.065)	1.107 (1.053–1.165)	1.176 (1.119–1.236)

Legend: ref. = reference group

The frequent manual handling of heavy loads and working under climatic workloads are associated with a slightly increased prevalence of neck and shoulder pain. Similar to the results for arm pain, weekly working hours are negatively associated with self-reported arm pain. Women have a higher prevalence of neck and shoulder pain than men. Age is associated with neck and shoulder pain (Table 4).

Without being split into specific work postures, frequently working in awkward body postures is associated with a slight increase in the prevalence of neck and shoulder pain of nearly 12% (PR 1.12, CI 1.06–1.19) (see additional material, Additional Table 2).

Without splitting the category “often works in awkward postures” into specific work positions, the estimated prevalence of neck and shoulder pain within the last 12 months based on the fully adjusted Model #5 is 56.4% (CI 53.9 - 59.0%) for employees who “often” work in awkward postures. The adjusted prevalence of neck and shoulder pain for subjects who reported that they “sometimes”, “rarely” or “never” work in awkward postures has been estimated at 51.3% (CI 48.9 - 53.8%), 49.5% (CI 47.2 - 51.8%) and 50.2% (CI 48.5 - 52.1%), respectively. Additional Table 2 (Additional material) provides the estimates of the regression model to which the above-mentioned estimations of the adjusted prevalence refer.

## Discussion

The study aims to specify the current prevalence of exposure to work in awkward postures and overhead work based on the German *2018 BIBB/BAuA Employment Survey*. A second objective is to identify occupational groups (jobs) that are more often exposed to overhead work as well as to work in awkward postures. Additionally, the study intends to demonstrate the association between working in awkward postures, especially overhead work, and the occurrence of arm pain, and to provide the adjusted prevalence of such self-reported symptoms.

In summary, the study has shown that 12.6% of participants indicated that they are “often” exposed to awkward postures at work. Nearly 5.0% reported that they often perform overhead work. Most of these employees work in agricultural, unskilled and skilled manual occupations. In general, men are more often exposed to work that results in awkward body postures. The analysis revealed an association between the self-reported frequency of overhead work and arm pain (“often”: PR 1.18, CI 1.04–1.34; “sometimes”: PR 1.19, CI 1.05–1.35; “rarely”: PR 0.99, CI 0.87–1.13; “never”: ref.)

The *2018 BIBB/BAuA Employment Survey* is a large-scale, representative, and cross-sectional-study. Therefore, we cannot derive causal inference. The interpretation of the associations between (self-reported) work in awkward body postures and the prevalence of arm pain or neck and shoulder pain must consider this aspect. Another aspect in this context is that the healthy worker effect could bias the analysis [6]. There is also the possibility that the results were affected by response bias and recall bias as all data is based on self-reported information. Workers with MSD pain might overestimate their exposures compared to workers who are asymptomatic. For example, based on a systematic review Coenen et al. 2019 [40] reported an increased occurrence of neck and upper extremity symptoms correlated with greater exposure to screen work. This effect was rather inconsistent and associated with weaker risks when screen work was assessed by software recordings compared to self-reports.

The weighted total sample of the *2018 BIBB/BAuA Survey of Employed Persons*, with *n* = 20,012 participants, is representative of the German labor force [24]. The weighting considered age, gender, household size, marital status, job position, nationality, and state of residence. For the present analyses, however, we used a subsample that was restricted with respect to age and weekly working hours. Therefore, weighting was not applicable. As a consequence, the representativeness of our subsample is to be regarded as limited.

Nevertheless, the aim of a cross-sectional study is to derive prevalence. Reporting a crude prevalence of the outcome stratified by the exposure of interest could be biased if main confounders such as age or gender are unequally distributed. This study provides estimations on the prevalence of arm pain based on the regression model and considering main confounders. The reported prevalence can be regarded as an adjusted estimate of actual prevalence in the exposure groups. The adjusted prevalence for arm pain stratified by the self-reported frequency of overhead work has been derived to be between 16 and 19%, while the unadjusted prevalence lies between 12 and 36%. We have to consider that all non-work and work-related covariates significantly contributed to the measured effect in the final model and reduced the estimate for overhead work seen in the crude model. This underlines the necessity for adjustment if an association between overhead work and the health complaints in the upper extremities is analyzed. Otherwise the effect would be overestimated.

The analysis is a secondary data analysis of the *2018 BIBB/BAuA Employment Survey*. We need to consider that the survey was primarily designed to answer other questions; thus, the applied adjustment for relevant covariables has been limited to available variables. Body weight or height, smoking, non-work related psychological stress, and vibration have not been considered within the *2018 BIBB/BAuA Employment Survey* and could not be implemented in the regression analyses. Future *BIBB/BAuA Employment Surveys* should include such covariates.

One advantage of the *2018 BIBB/BAuA Employment Survey* is that it covers a large study population of 20,012 employees. Out of 47.4% reachable employees, (*n* = 42,188) participated. Unfortunately, an analysis of non-responders is not available. Despite the limitations, the study is based on one of the largest employment surveys in Germany.

The exposure prevalence of 12.7% for employees who “often” work in awkward postures was lower than in previous surveys conducted in 2012 (16.6%) and 2006 (14.3%). The difference could be related to differences in inclusion criteria. While the dataset of this study included subjects who work more than 35 h per week, the prevalence reported in previous surveys in 2006 and 2012 included employees who worked less than 35 h per week [41, 42]. The present study shows an exposure prevalence of employees who often perform overhead work of 5.0%. Previous *BIBB/BAuA Employment Surveys* did not include the variable of overhead work. Thus, comparisons or time-trend analyses are not possible with regard to this type of work in awkward postures [41, 42].

The *2018 BIBB/BAuA Employment Survey* derived information about work performed in awkward body postures in more detail than former surveys, however, only in cases in which a subject responded that they “often” worked in awkward postures. Dealing with such complex structured exposure variables is difficult. In future surveys, the variables of “works in awkward postures” and “overhead work” should be assessed separately as has been done in comparable employment surveys, for example in Denmark or Norway [26]. This modification may make it possible to gain more information about overhead work and to simplify the statistical analysis. Another limitation is that the answer categories (“never”, “rarely”, “sometimes”, and “often”) employed here cannot be linked to the duration or an absolute frequency per working day. No further assistance with interpretation of these categories was given to the surveyors. Alternatively, the surveys in other countries (Denmark, Norway, and Spain) use fractions of the working day [26] or retrospective duration of jobs requiring physical workload [43].

The possibility of comparing the considered outcomes of “self-reported arm pain” or “neck and shoulder pain” (in the last 12 months) to other studies, for example Luime et al. 2004 [29], is limited. A more specific outcome should be restricted simply to “shoulder pain”. The questionnaire of the *2018 BIBB/BAuA Employment Survey* differentiates two regions: “neck and shoulder” and “arm”. The crude prevalence of pain in the “neck and shoulder” region is extremely high (48.4%). The gradient of prevalence over the categories of the self-reported frequency of work performed in awkward postures is low (46 to 61%) compared to arm pain (12 to 36%). The adjusted regression analysis using “neck and shoulder pain” as an outcome has provided only an extremely weak association with regard to work frequently performed in awkward postures (fully adjusted model: PR 1.12, CI 1.05–1.19) (Additional Table 2) and no association to overhead work (fully adjusted model: PR 1.04, CI 0.96–1.12) (Table 4). Therefore, we have decided to use “pain in the arm region” as the more specific outcome with regard to exposure to “overhead work”. A second issue is that the wording of the question regarding pain or symptoms is conditional to an association to work (“while or after work”). At this point, we suggest a more specific and unconditional operationalization of the health outcomes used in the *BIBB/BAuA Employment Survey*.

The crude tabulations show that subjects who often work overhead had a nearly threefold increased prevalence of arm pain in the last 12 months. After adjusting for relevant covariables, a significant association between overhead work and arm pain remains. Participants who often work overhead had an 18% higher prevalence (PR 1.18, CI 1.04–1.34) of arm pain in the last 12 months than employees who are not exposed to such postures. The results of this study have confirmed the expected association between overhead work and arm pain. Previous studies have demonstrated that overhead work is a risk factor for shoulder pain [4, 6–12, 14]. In a recent systematic review, Wærsted et al. 2020 [20] provided similar effects in studies using questionnaires or interviews to assess work with elevated arms in relation to shoulder pain in the last 12 months based on cross-sectional studies and case-control studies. It should be noted that working above shoulder level is linked not only with discomfort, but also with specific shoulder diseases. This could be shown in a recently published systematic review for cumulative exposure to various physical occupational demands such as working above shoulder level, repetitive movements, forceful work, and hand-arm vibrations [44].

In summary, despite the increasing technological and digital transformation process, many employees are still exposed to awkward postures and overhead work. Technical and ergonomic solutions to reduce the workload caused by overhead work are available but are rarely used. Advanced technical support, such as the use of exoskeletons, is still in development and limited to specific conditions. Considering the demographic change, degenerative musculoskeletal disorders are still expected to become an increasing problem. This may lead to an increase in health inequality, long-term sick leave, early retirement and difficulty in continuing to work in jobs with a high physical workload. In order to ensure jobs with a high physical workload continue to be attractive and to avoid economic losses, already existing prevention strategies have to be reconsidered and improved.

## Conclusions

The results demonstrate that in the German labor force especially people employed in industrial and manufactural sectors are exposed to awkward postures and overhead work, respectively. In general, men are more often exposed to work that results in awkward postures than women. Overhead work should be considered a risk factor for the occurrence of shoulder pain and arm pain. Finally, methodological improvements to the German monitoring of surveys should be discussed.

## Supplementary Information

**Additional file 1: Table 1.** Prevalence ratios (PR) with 95% confidence interval considering pain in the arm region. Prevalence ratios for important variables without splitting the category “often works in awkward postures” considering pain in the arm region.

**Additional file 2: Table 2.** Prevalence ratios with 95% confidence interval considering pain in the neck and shoulder region. Prevalence ratios for important variables without splitting the category “often works in awkward postures” considering pain in the neck and shoulder region.

**Additional file 3: Figure 1.** Flowchart of the construction of the dummy-variables for working in awkward postures used in the regression analyses.

## Data Availability

The complete dataset of the 2018 *BIBB/BAuA Employment Survey* supporting the conclusions of this article is available as a scientific-use-file (no. ZA7574) and can be requested at „BIBB – Bundesinstitut für Berufsbildung - Forschungsdatenzentrum “(post office box 201264; 53142 Bonn; Germany; fax number: + 49 – (0)228–107 – 2020); https://www.bibb.de/de/120401.php). After approved application the dataset will be available as an ftp-download.

## Abbreviations

BAuA: Federal Institute for Occupational Safety and Health
BIBB: Federal Institute for Vocational Education and Training
CI: Confidence interval
EU-OSHA: European Agency for Safety and Health at Work
MSD: Musculoskeletal disorders
PR: Prevalence ratio
SD: Standard deviation
