# Supplementary material for: Association between working in awkward postures, in particular overhead work, and pain in the shoulder region in the context of the 2018 BIBB/BAuA Employment Survey
Source: BMC Musculoskelet Disord. 2021 Jul 15;22:624. doi: 10.1186/s12891-021-04482-4 (PMC8283940; doi:10.1186/s12891-021-04482-4)
Supplement: Supplementary file 1 — Additional file 1: Table 1. Prevalence ratios (PR) with 95% confidence interval considering pain in the arm region. Prevalence ratios for important variables without splitting the category “often works in awkward postures” considering pain in the arm region. [file 12891_2021_4482_MOESM1_ESM.pdf]

Additional Table 1: Prevalence ratios (PR) with 95 % confidence interval considering **pain in the arm region** (n = 14,327)

	**Prevalence ratios for arm pain after adjusting for gender, age, weekly working hours and working conditions (Model #5)**			
**Age**	Per year	1.020<br>(1.017 - 1.023)		
**Gender**	Women	1.449<br>(1.349 - 1.556)		
**Weekly working hours**	Per h	0.987<br>(0.982 - 0.992)		
**Psychosocial workload (score)**	Per unit	1.011<br>(1.008 - 1.014)		
	Never	Rarely	Sometimes	Often (without splitting)
**Works in awkward postures**	1 (ref.)	0.955<br>(0.848 - 1.077)	1.169 (1.037 - 1.317)	1.357<br>(1.210 - 1.522)
**Manual lifting of heavy loads**	1 (ref.)	1.053<br>(0.939 - 1.180)	1.146 (1.006 - 1.306)	1.471<br>(1.303 - 1.660)
**Manual handing operations**	1 (ref.)	1.010<br>(0.866 - 1.179)	1.125 (0.978 - 1.293)	1.670<br>(1.493 - 1.868)
**Climatic workload**	1 (ref.)	1.056<br>(0.931 - 1.197)	1.283<br>(1.153 - 1.428)	1.630<br>(1.475 - 1.801)

Legend: ref.: reference group
