# Supplementary material for: Association between working in awkward postures, in particular overhead work, and pain in the shoulder region in the context of the 2018 BIBB/BAuA Employment Survey
Source: BMC Musculoskelet Disord. 2021 Jul 15;22:624. doi: 10.1186/s12891-021-04482-4 (PMC8283940; doi:10.1186/s12891-021-04482-4)
Supplement: Supplementary file 2 — Additional file 2: Table 2. Prevalence ratios with 95% confidence interval considering pain in the neck and shoulder region. Prevalence ratios for important variables without splitting the category “often works in awkward postures” considering pain in the neck and shoulder region. [file 12891_2021_4482_MOESM2_ESM.pdf]

Additional Table 2 Prevalence Ratio with 95 % confidence interval considering **<u>pain in the neck and shoulder region</u>** (n = 14,327)

	**Prevalence ratios for neck/shoulder pain after adjusting for gender, age, weekly working hours and working conditions (Model #5)**			
**Age**	Per year	1.004 (1.002 - 1.005)		
**Gender**	Women	1.571 (1.518 - 1.625)		
**Weekly working hours**	Per h	0.993 (0.990 - 0.995)		
**Psychosocial workload (score)**	Per unit	1.010 (1.009 - 1.012)		
	Never	Rarely	Sometimes	Often (without splitting)
**Work in awkward postures**	1 (ref.)	0.984 (0.930 - 1.041)	1.021 (0.963 - 1.083)	1.122 (1.059 - 1.188)
**Manual lifting of heavy loads**	1 (ref.)	1.006 (0.957 - 1.057)	1.005 (0.946 - 1.067)	1.109 (1.048 - 1.175)
**Manual handing operations**	1 (ref.)	0.963 (0.904 - 1.026)	0.947 (0.891 - 1.007)	1.016 (0.968 - 1.066)
**Climatic workload**	1 (ref.)	1.004 (0.949 - 1.063)	1.104 (1.050 - 1.161)	1.173 (1.116 - 1.233)

Legend: ref.: reference group
