# Supplementary material for: Association between working in awkward postures, in particular overhead work, and pain in the shoulder region in the context of the 2018 BIBB/BAuA Employment Survey
Source: BMC Musculoskelet Disord. 2021 Jul 15;22:624. doi: 10.1186/s12891-021-04482-4 (PMC8283940; doi:10.1186/s12891-021-04482-4)

Additional Figure 1: Flow-chart of the construction of the dummy-variables for working in awkward postures used in the regression analyses.

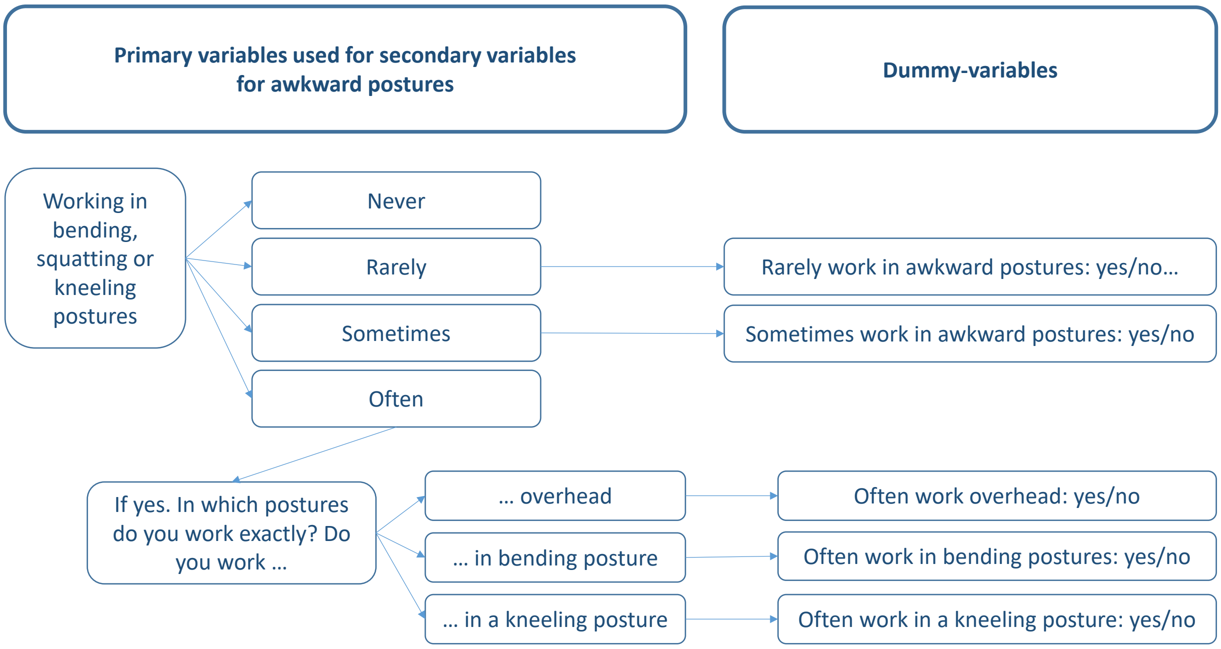

Supplement: Supplementary file 3 — Additional file 3: Figure 1. Flowchart of the construction of the dummy-variables for working in awkward postures used in the regression analyses. [file 12891_2021_4482_MOESM3_ESM.pdf]
